# 3D Printing Materials Mimicking Human Tissues after Uptake of Iodinated Contrast Agents for Anthropomorphic Radiology Phantoms

**DOI:** 10.3390/biomimetics9100606

**Published:** 2024-10-08

**Authors:** Peter Homolka, Lara Breyer, Friedrich Semturs

**Affiliations:** 1 Center for Medical Physics and Biomedical Engineering, Medical University of Vienna, 1090 Vienna, Austria; 2 Department of Biomedical Imaging and Image-Guided Therapy, Medical Imaging Cluster (MIC), Medical University of Vienna, 1090 Vienna, Austria

**Keywords:** radiology phantoms, medical imaging, contrast-enhanced mammography, computed tomography, quality control, iodine contrast, anthropomorphic phantoms, tissue mimicking materials, additive manufacturing, 3D printing

## Abstract

(1) Background: 3D printable materials with accurately defined iodine content enable the development and production of radiological phantoms that simulate human tissues, including lesions after contrast administration in medical imaging with X-rays. These phantoms provide accurate, stable and reproducible models with defined iodine concentrations, and 3D printing allows maximum flexibility and minimal development and production time, allowing the simulation of anatomically correct anthropomorphic replication of lesions and the production of calibration and QA standards in a typical medical research facility. (2) Methods: Standard printing resins were doped with an iodine contrast agent and printed using a consumer 3D printer, both (resins and printer) available from major online marketplaces, to produce printed specimens with iodine contents ranging from 0 to 3.0% by weight, equivalent to 0 to 3.85% elemental iodine per volume, covering the typical levels found in patients. The printed samples were scanned in a micro-CT scanner to measure the properties of the materials in the range of the iodine concentrations used. (3) Results: Both mass density and attenuation show a linear dependence on iodine concentration (R^2^ = 1.00), allowing highly accurate, stable, and predictable results. (4) Conclusions: Standard 3D printing resins can be doped with liquids, avoiding the problem of sedimentation, resulting in perfectly homogeneous prints with accurate dopant content. Iodine contrast agents are perfectly suited to dope resins with appropriate iodine concentrations to radiologically mimic tissues after iodine uptake. In combination with computer-aided design, this can be used to produce printed objects with precisely defined iodine concentrations in the range of up to a few percent of elemental iodine, with high precision and anthropomorphic shapes. Applications include radiographic phantoms for detectability studies and calibration standards in projective X-ray imaging modalities, such as contrast-enhanced dual energy mammography (abbreviated CEDEM, CEDM, TICEM, or CESM depending on the equipment manufacturer), and 3-dimensional modalities like CT, including spectral and dual energy CT (DECT), and breast tomosynthesis.

## 1. Introduction

Phantoms serve as physical [1] or computerized models [2,3] that simulate human tissues and organs in various applications from hardware evaluation, optimization of procedures to system testing and quality assurance. These specialized objects also play a crucial role in various aspects of medical imaging, including research, development, and regular quality control, all serving vital roles in patient care optimization. Especially when the phantom design involves realistic structures and details, additive manufacturing and 3D printing are indispensable, offering the ability to produce phantoms with patterns with a high degree of realism [4,5,6]. Setting it apart from a simple test object, a phantom is defined as a model of the human body or body part designed to replicate specific features or interactions relevant to the specific application, e.g., a medical imaging procedure. 3D-printed phantoms are used across all diagnostic and therapeutic radiologic modalities, including ultrasound, MRI, CT, mammography, nuclear medicine, and radiation therapy [5,7,8]. The primary purpose of phantoms in medical imaging with ionizing radiation and radiation therapy is to evaluate and optimize imaging devices and procedures, facilitating virtual trials, managing protocols, assessing diagnostic performance, conducting dosimetry studies [9], and ensuring that radiation safety standards are met [10,11].

Phantoms can range from simple to highly complex, depending on their intended application. Simple phantoms include basic slabs or stylized objects that mimic typical patient attenuation in medical X-ray fields, while anthropomorphic phantoms are complex task-specific models designed to study the effects of dose level variation, radiation quality, or system specifications on diagnostic performance. Since no real anthropomorphic phantom that mimics all the relevant properties is yet available, usually, procedure/task specific pseudo-anthropomorphic phantoms are designed and used, mimicking the most relevant properties with a wide range of finesse. Digital phantoms serve as computerized models used for dosimetry, in silico testing, and virtual imaging trials [2,12,13]. Another dedicated family of phantoms is represented by calibration phantoms, which are utilized for quantitative imaging to calibrate results, and/or perform accuracy tests of clinical data [14,15,16,17,18,19].

The construction of phantoms involves materials that mimic the properties of human tissues or well-defined materials in relation to the specific imaging modality. For X-ray imaging, materials must replicate the modification of radiation fields caused by absorption and scattering in body tissues [20,21].

As imaging technologies advance, there is a growing need for more sophisticated “super phantoms” that can reproduce in vivo imaging features with high fidelity, combine digital and physical elements for comprehensive testing, serve as ground truth for various properties and imaging biomarkers, and facilitate the development and validation of machine-learning algorithms in medical imaging.

Additive manufacturing, particularly 3D printing, has revolutionized phantom production by offering flexibility in design and cost efficiency, and an accurate representation of the anatomy and tissue properties [22]. One of the most significant constraints associated with the 3D printing of radiographic phantoms, test objects, and calibration standards is the restricted range of printable polymers. In addition, these commercially available materials are usually designed for different applications, with optimization mainly occurring in terms of mechanical properties, user-friendliness or the visual appearance of the printed objects. A variety of studies has been undertaken to test and establish the radiologic equivalence of available printing materials with tissues and standard materials like liquid water for a variety of X-ray photon energies, from mammography to CT and therapeutic photon energies in radiotherapy [23,24,25,26,27,28,29,30,31]. Nevertheless, challenges remain in developing 3D-printable materials that accurately mimic tissue properties across a wide range of imaging modalities and energy levels.

Iodine plays a key role in X-ray imaging as a contrast agent, primarily due to its high atomic number and K-edge at 33.2 keV closely matching the energy range of X-rays used in diagnostic imaging, resulting in strong X-ray attenuation due to the photoelectric effect. Iodine-based contrast agents are used in a variety of imaging modalities, including computed tomography (CT), fluoroscopy, angiography, and interventional radiology. By combining images taken at different photon energies in DECT (dual energy CT) [32] and CEDEM (contrast enhanced dual energy mammography [33]), iodine-enhanced tissues or lesions can be isolated from the anatomical background and/or iodine uptake quantified.

Typical iodine uptake in CT examinations depends on the contrast medium application protocol, as well as patient-related factors, such as age, gender, and body mass index [34]. In healthy subjects, typical iodine concentrations in the tissues of interest in standard contrast-enhanced CT scans range from typically 1 to 6 mg iodine per ml tissue [34]. Depending on the scan protocol (kV used in standard scans, or keV reconstruction energy in pseudo-monochromatic reconstructions), the voxel value increases by typically >20 (140 kV) to >45 HU (80 kV) per mg Iodine/mL, with a value of approximately slightly above 25 HU per mg/mL iodine in standard scan conditions (120 kV) [35]. Thus, the range of iodine concentrations in typical phantoms is expected to be in the range of <1 to a few percent of iodine.

In contrast to CT, where iodine content is quantified volumetrically (in mg/mL), in 2D imaging modalities (like general radiography, dual energy mammography except breast tomosynthesis, and fluoroscopy including angiography), only total attenuation is represented in the pixel values. Therefore, iodine content needs to be specified as the iodine area weight (also referred to as area density, corresponding to the product of volume density and thickness) typically provided in mg/cm^2^. While in angiography and interventional cardiology, high iodine concentrations are used to visualize dynamic processes that are best replicated in phantom models by using liquid iodine solutions, contrast-enhanced mammography (CEDEM) represents a modality where very accurate low iodine concentrations in phantoms are necessary. The field of applications in CEDEM ranges from system testing and the optimization of image-processing algorithms [36] to regular quality control. Necessary iodine concentrations for CEDEM phantoms typically range from 0.25 to 2 mg/cm^2^ iodine [19,37]. If these phantom details were to be printed with 1 mm of thickness, the corresponding area weights necessitate an iodine concentration (*w*/*w*) of approximately 0.2 to 1.6 mg/mL. In patient images, iodine enhancement in lesions was shown to exceed 0.5 mg/mL iodine with a peak at 1 mg/mL in the great majority of clinical cases, with only a few single cases exhibiting enhancements greater or equal to 3 mg/mL [38].

Phantoms containing iodine in appropriate concentrations thus are not only important for research and development, but will find their application in the regular quality assurance and continuing optimization of both acquisition protocols and imaging devices. Most of these phantoms use cavities filled with liquid solutions containing the desired iodine concentration(s) [39,40,41,42,43]. However, solid iodine contrast phantoms offer several advantages over traditional liquid-filled phantoms. While liquid-filled phantoms or phantom inserts allow for adjustable iodine concentrations, they present challenges such as potential evaporation, leakage, and bubble formation during refilling. These issues can limit long-term stability and complicate use when concentration adjustments are not required.

To address these limitations, researchers have explored solid phantom designs incorporating iodinated materials. One approach is to embed iodobenzene in an epoxy resin matrix [44]. Production involves handling toxic and potentially difficult-to-access chemicals (like iodobenzene). Another option is to incorporate iodine (as Iohexol powder) into paraffin-based formulations that mimic tissue- and water-equivalent materials [45]. Epoxy resin-based phantom materials have a long history in tissue- and water-equivalent materials for imaging and radiotherapy applications [46]. However, the production method requires careful manufacturing techniques to prevent air bubble formation and sedimentation, which are typically achieved by vacuum mixing and the control of viscosity. When produced under optimum conditions, both of these, epoxy- and paraffin-based phantoms proved effective for various imaging applications, including CT and radiotherapy. However, none of these materials can be used for additive manufacturing in combination with 3D printing.

By providing multiple fixed iodine concentrations in a single stable unit, solid iodine phantoms provide a convenient and reliable tool for imaging research and quality assurance [19]. These phantoms eliminate concerns about evaporation, leakage, and air entrapment associated with liquid-filled designs, while still allowing the simultaneous imaging of multiple iodine concentrations.

The aim of this work was to develop, test, and verify a method to produce easily available printing materials with exactly definable iodine concentrations, that are chemically stable, cheap and readily available, can easily be produced in average medical research settings, and can be printed with consumer grade 3D printers.

## 2. Materials and Methods

Commercially available 3D-printing resins, which are aimed at the mass market of hobbyists and are available in a wide range on internet marketplaces such as Amazon, have been doped with an iodine contrast agent commonly used in diagnostic X-ray imaging and printed on a consumer grade resin printer to produce samples with appropriate iodine concentrations mimicking those of tissues after contrast agent administration. Samples were then evaluated in a micro-CT scanner to measure the X-ray attenuation dependent on iodine concentrations. The reproducibility and stability of the process was evaluated by reprinting samples with a different batch of the printing resin.

### 2.1. Doping of Printing Polymers

Standard 3D-printing polymers targeting professional and hobbyist users were obtained from major online marketplaces. Resins were mixed with 5 to 10 percent contrast media and allowed to settle overnight in a first test run, and for extended time periods in a second test round. The resins that either gelled as a reaction to the contrast agent introduced or where the iodine complex in the contrast agent precipitated as a white powder were discarded.

As contrast agent, the formulation with the highest available iodine concentration used at our university hospital was chosen (Iomeron 400, Bracco Imaging S.p.A, Milan, Italy). It contains Iomeprol (C_17_H_22_I_3_N_3_O_8_) with 400.0 mg iodine per mL solution. From the chemical composition, the elemental iodine content in Iomeprol is calculated as 48.992%. To calculate mixing ratios with the resin, the mass density of the liquid contrast agent was measured using a self-calibrating analytical laboratory balance (Sartorius, Göttingen, Germany) and a calibrated pycnometer with a nominal volume of 25.131 mL. The volume of the pycnometer was verified by measuring deionized water at 22 °C, resulting in a correction of 0.1%. The density of the contrast agent was determined at the laboratory temperature of 22 °C as 1.450 ± 0.0014 g.cm^−3^. Using this density and the elemental iodine content of the contrast agent, the iodine content translates from 400.0 mg/mL to 0.2759 g iodine per g Iomeron 400. Using these numbers, the mixing rations of the base resin with the contrast agent were calculated. The doping process in the chemical lab was performed by weighing the desired amount of resin into 500 mL light–tight wide neck PE laboratory bottles and adding the liquid contrast agent with a Pasteur pipette on the balance with sub mg accuracy. Then, the bottles were closed and agitated by hand to ensure the appropriate mixing of the two liquids.

### 2.2. Determination of Mass Density of Printed Samples

In volumetric imaging modalities like CT, iodine concentrations are specified in mg iodine per ml (or cm^3^). However, the volume of uncured resin does not exactly equal the volume of the cured resin, since polymerization during the printing process is associated with (minimal) volume loss due to shrinkage. In addition, resin/contrast agent mixtures are prepared and specified as iodine per weight (% *w*/*w*) rather than per volume, because mixing components in percent by weight implies a much smaller uncertainty than mixing as mass per volume (% *w*/*v*) in a research laboratory setting.

To determine the mass density as a function of iodine content, three cylinders each with a 1.5 cm diameter and a 2 cm height were printed with iodine concentrations of 0, 1, 1.5, and 2% iodine by weight, corresponding to 0 to 7.250% *w*/*w* of liquid Iomeron 400, respectively. To calculate the mass densities, the weight was determined using a self-calibrating analytical laboratory balance. To calculate the volume of the cylinders, the height and diameter were measured using a digital caliper (Mitutoyo, Kawasaki, Japan). Measurements were taken 3 times at different positions (height) and 6 times (diameter) for each sample at perpendicular angles and different heights (top/middle/bottom), then averaged.

### 2.3. Samples for Measurement of Attenuation vs. Iodine Content

Since the samples printed for the determination of mass density (up 2% iodine content *w*/*w*) printed identically to the undoped resin, the iodine concentration was increased to 2.5% (corresponding to 9.063% Iomeron 400), and further to 3% iodine (10.875% Iomeron 400), respectively. Cylindrical samples with three sections, each 10 mm long, and 8, 5, and 3 mm in diameter, respectively, were printed with all the iodine concentrations studied. For the determination of the dependence of the measured attenuation on iodine content in the micro-CT scanner, the rod sections with 8 mm diameter and 10 mm length were used. These were produced in 2 printing runs; in the first printing run, the samples for scanning were printed together with the larger samples used for the determination of mass density (0 to 2% iodine content). In a second run, 2% iodine was repeated using a different (re-ordered) batch of the base resin to test the reproducibility and 2.5 and 3% iodine containing samples were added. For each concentration except 2% iodine, two samples were scanned in the micro-CT scanner. For 2% iodine, 4 samples were used; two from each printing run were scanned to compare the results from the two different mixing and printing cycles using two different batches of resin.

Samples were printed on a consumer-grade (M)SLA printer (Elegoo Mars 4 Ultra, Elegoo Inc., Shenzhen, China), employing a 7-inch 9K Mono LCD screen with a resolution of 8520 × 4320 pixels. Printing parameters were determined with the undoped and doped resin, applying the Photonsters Validation Matrix V2 [47]. However, no changes in the exposure parameters between the doped and the stock resin were found; thus, exposure parameters for the stock resin were used.

After printing, the samples were spray-cleaned with a sequence of 100% IPA (isopropyl alcohol) followed by 30% IPA and tap water, then washed with liquid hand soap to remove the last traces of uncured resin, followed by rinsing off with deionized water. After drying for a minimum 24 h, the samples were post-cured in a curing chamber (Wash and Cure, Anycubic, Shenzhen Anycubic Technology Co. Ltd., Shenzhen, China) for 3 min.

Printing models were designed in Fusion 360 (Autodesk Inc., San Francisco, CA, USA) and STL files were sliced with Chitubox V1.9.5 (Shenzhen CBD Technology Co. Ltd., Shenzhen, China).

### 2.4. CT Scanning of Iodine Samples

**Figure 1:**
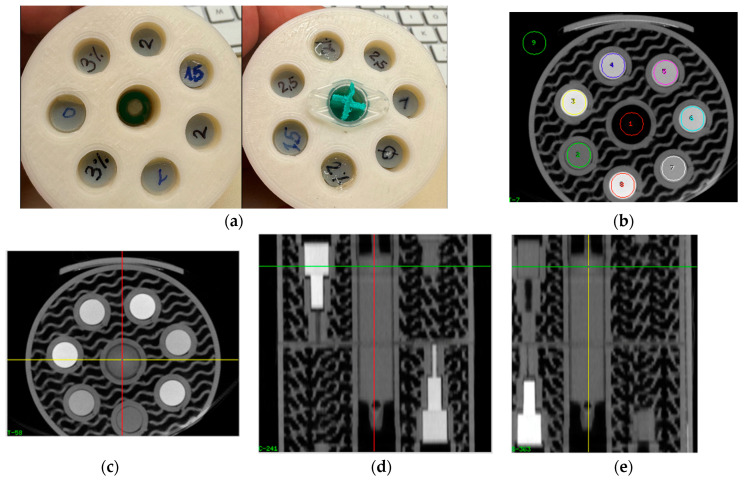
(**a**) PLA phantom of 6 cm diameter used for scanning of printed samples. View from above and below showing individual samples, and a 2 mL syringe in center filled with deionized water. (**b**) Single slice of the micro-CT image volume, indicating placement of ROIs used for determination of voxel values. (**c**–**e**): Transverse, coronal, and sagittal reformat of CT volume showing samples inside phantom. (**c**) Transversal reformat through section showing 6 cylinders with 8 mm diameter used for determining voxel values with iodine concentrations from 0 to 2.5% iodine, and water in the center. In (**d**,**e**) the water-filled syringe is shown centrally. Note: phantom sections containing thinner rods (5 and 3 mm in diameter) seen in coronal and sagittal reformats were not used in this work.

For scanning, the printed samples were mounted into cylindrical phantoms 6 cm in diameter and 7 cm in length, respectively (Figure 1, panel (a)). This phantom was printed with a PLA filament (ecoPLA Tough transparent, 3DJake, Niceshops GmbH, Paldau, Austria) with an FDM printer (Flsun V400, Zhengzhou Chaokuo Electronic Technology Co., Ltd., Zhengzhou, China) using a 0.8 mm nozzle and 30% gyroid infill, guaranteeing the structural stability of the phantom while allowing for minimal X-ray attenuation in the scanning process, still ensuring correct working of the beam-hardening correction. In the center, a 2 mL syringe filled with deionized water for the comparison of image noise and normalization of CT voxel values was placed.

CT scanning was performed in a Siemens Inveon Trimodality scanner (Inveon Trimodality imaging system, Siemens Healthineers, Erlangen, Germany), applying a CCD detector combined with a variable focal spot X-ray source, producing an image volume of 1024 × 1024 × 1024 isotropic voxels with 0.0975^3^ mm^3^ size. The scan was performed with 70 kV and 500 µA tube current in Hires Mode with a static table. The reconstruction was performed using the Feldkamp cone beam algorithm, employing a Shepp filter with a cut-off at the half-Nyquist frequency. Since image values are not provided in Hounsfield units (HU) with zero indicating no attenuation rather than water, voxel values of water were measured in the water-filled syringe in the center of the phantom. Additionally, a ROI (number 9 in Figure 1b) outside the phantom was placed in the air to verify the scaling of voxel values. The voxel value histogram measured in the air exhibited a strong peak at zero, followed by a half-sided Gaussian distribution corresponding to the normalization of the voxel values to zero at no measured attenuation.

### 2.5. Data Analysis

The micro-CT image volume stored as 16-bit integer values was loaded into the Analyze image processing software package [48] (Analyze 12.0), Biomedical Image Resource (Mayo Clinic, Rochester, NY, USA) as raw data and reformatted to a slice thickness of 1 mm for evaluation. Voxel values in the samples were measured in the image slices containing the cylindrical samples with an 8 mm diameter. The sample sections of 5 and 3 mm in diameter were not used, since no beam-hardening effects from the iodine attenuation were seen for all the iodine concentrations studied, that would have necessitated a reduction of the iodine content in the scan cross-section to derive exact attenuation measurements.

To avoid partial volume effects, circular regions of interest with a 65 voxel (6.3 mm) diameter slightly smaller than the cylinders were applied (Figure 1b), and the top and bottom slices were omitted. Depending on the sample, 8 or 9 artefact free slices remained, and the voxel values of the individual slices were averaged. ROIs were also placed into the filled syringe at the center to measure the voxel value of water for comparison. In addition to the average values, the STDs of average voxel values in the individual slices were also determined and uncertainties were calculated.

Data evaluation was performed in Microsoft Excel, Ver.16.88 Standard for Mac 2021 (Microsoft Corp., Redmont, WA, USA).

## 3. Results

### Mass Density of Printed Iodine Samples and Conversion to % w/v

**Figure 2:**
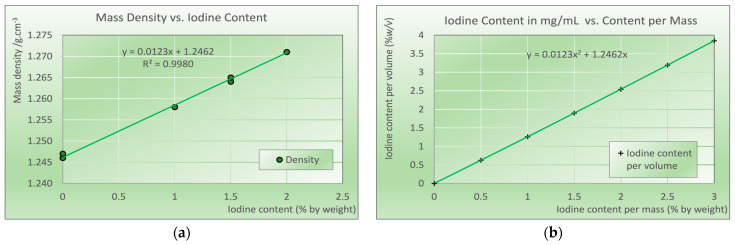
(**a**) Mass density measured in the test cylinders as a function of the iodine content. Points shown correspond to measurements from individual cylinders; (**b**) conversion of iodine content per weight (% *w*/*w*) to iodine content per volume (% *w*/*v*) as used in tomographic imaging. Points shown correspond to the calculated values using linear regression from (**a**).

Mass densities determined from the test cylinders 2 cm in height and 1.5 cm in diameter are shown in Figure 2. A linear dependence (R^2^ = 0.9980) of density versus iodine content demonstrates the density increase with increasing iodine content due to the higher mass density of contrast media compared to the base resin. Using the equation derived from the regression analysis, the iodine content per mass translates to the iodine content per volume.

**Figure 3:**
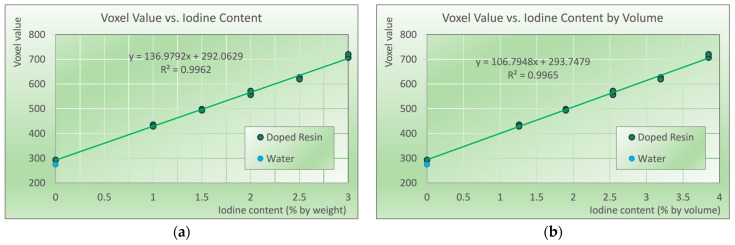
Voxel values of the samples printed with iodine concentrations from zero (base polymer) to 3% elemental iodine (*w*/*w*) corresponding to 3.85% *w*/*v*. Blue data point at zero iodine content corresponds to the voxel value of deionized water. 1σ error bars are smaller than symbol size. (**a**) Voxel value vs. iodine content by weight, and (**b**) vs. iodine content per volume, converted according to Figure 2b.

Figure 3 indicates the voxel values measured in the micro-CT scanner vs. the iodine content by weight (panel (a)), and by volume (panel (b)). The linear regression of voxel values versus iodine content per volume indicates an almost perfect (R^2^ = 0.9965) linear dependence of iodine content and attenuation, indicating an accurate and stable production process. The voxel value of the demineralized water is shown as a blue data point in the figure. Owned to the higher mass density of the printing resin, the attenuation of the resin is slightly above water. In the printed samples, no indications of inhomogeneities were present; the average standard deviation of voxel values within each sample was 5.63 and thus not larger than in the water in the centrally placed syringe (6.65).

With 2% *w*/*w* iodine content, four cylinders (two each) were produced with two different batches of resin, with different production dates indicated on the resin bottles in independent production cycles several weeks apart. The average voxel values measured in the individual CT slices in the cylinders produced with the different batches are not statistically different (*p* > 0.40, Cohen’s d < 0.15). Thus, the production process is classified as stable and independent of the resin batch.

## 4. Discussion

Standard printing polymers, used for either fused deposition modeling (FDM) printers printing with solid filaments, stereolithography (SLA, including masked SLA or MSLA, also termed LCD printing), digital light processing (DLP), or polyjet technology, which utilizes liquid printing resins solidified in the printing process by irradiation with visible or UV light, can be doped with functional additives. Doping printing polymers involves adding specific functional substances to modify their properties for custom applications. In general-purpose and industrial 3D printing, examples include adding carbon nanofibers, nanotubes, or graphene to increase the electrical conductivity or mechanical strength, ceramic powders to increase the thermal resistance, flame retardants, or UV resistant agents. In medical-imaging applications, resins doped with radioactive tracers are used in nuclear medicine phantoms [49,50] mimicking patient tissue after the uptake of radioactivity. In X-ray imaging, several high Z materials are typically added to increase X-ray attenuation. Bismuth-doped ABS is suggested for radiation shielding or X-ray phantoms [51] mimicking different body tissues from soft tissues to bone. Printing iodine-doped ink with a consumer grade inkjet printer on paper and stacking up the printed sheets may not be considered 3D printing, but it represents another possibility for producing 3D phantoms with doped printing inks [52].

When doping printing polymers or resins with additives, there are several potential problems that need to be addressed. While it is relatively easy to mix solid polymer pellets or powders with the desired additives to produce custom filaments for FDM printing, this process usually requires industrial equipment such as mills and extrusion machines, and also requires larger batches. In most cases, these issues preclude the option to produce custom filaments for research groups involved in the development of phantoms in a medical environment [53].

Doping liquid resins with functional additives, on the other hand, can normally be done in a small lab without the need of specialized equipment. Historically, epoxy resin systems have been used to produce numerous phantom materials mimicking different body tissues very well by mixing the resins with mineral components like CaCO_3_, MgO, TiO_2,_ and other materials to compensate for the typically low effective atomic number of the base resin [46]. To mimic the X-ray attenuation properties of soft tissues and liquid water, the mass density of the resulting material needed to be lowered, which was typically achieved by adding gas-filled phenolic microballoons. The two main issues in the production of these phantom materials are the separation of additives, since the heavier powders tend to sink to the bottom and the microspheres tend to float. To avoid or at least minimize these separation effects, viscosity has to be controlled. This can be done by adding fine polymer powders like polyethylene. The other issue, especially since the viscosity was often increased to avoid sedimentation and the separation of the microballoons, is the trapping of air bubbles during the mixing process. These were, in a next step, removed in a vacuum chamber [46,54,55]. To avoid these issues, a production method based on mixing and sintering fine polymer powders with additives was developed [56,57]. It should be noted that these formulations, however, could easily be used—possibly with minor adaptions to the base polymers—for the production of radiologically tissue-equivalent filaments for FDM printing.

Modern 3D-printing resins for DLP/(M)SLA printers possess very low viscosity to allow fast printing with high spatial resolution. As a result, mixing solid powders into these resins tends to result in sedimentation. In standard prints, this is observed with color pigments and minimized by agitating the resin before and, in some printers, also during the printing process. While a minor issue in the additive manufacturing of functional prototypes or decorative items, the sedimentation of powders added to control X-ray attenuation will result in inhomogeneous phantoms exhibiting a gradient in X-ray attenuation properties, resulting in inaccurate and inconsistent properties prohibiting clinical use. To avoid the problem of separation, possible solutions include the use of dopants that either dissolve in the liquid resin, or are liquid themselves and chemically inert to the resin. In this work, it was shown that standard printing resins can be doped with liquid iodine solutions to avoid concentration inhomogeneities and inaccuracies due to separation issues from adding iodine compounds in powder form, like Iopamidol powder [19], without negatively influencing their printing properties. However, it needs to be noted that a verification of the homogeneous distribution of the additive—iodine, in this case—needs to be verified, as not all commercial 3D-printing resins allow the formation of a homogeneous mixture with aqueous iodine-containing solutions, such as X-ray contrast media, that will print appropriately.

During the development of these iodine-doped resins, we also encountered difficulties because suppliers changed the chemical formulations of the resins with new batches. This happened with the first resin identified as suitable for iodine contrast agent doping. The re-ordered resin did not form a homogeneous mixture with the contrast agent and gelled after a short time, preventing printing samples with accurate and homogeneously distributed iodine content. Therefore, each batch should be checked before printing phantoms or phantom parts. If this is verified, doping resins with liquid iodine contrast agents intended for patient use provides a very accurate and appropriate way of adding iodinated contrasts or details with very well-defined iodine content to custom phantoms.

### Limitations, Future Work, and Outlook

In this work, the resulting iodine concentrations have not been verified by independent measurements and rely on the accuracy of the iodine content in the contrast medium stated by the producer as 400.0 mg/mL. While extensive quality assurance is mandatory for medically used drugs like contrast agents, and the product data sheet specifies an accuracy better than ±0.05 mg/mL (400.0), larger uncertainties are involved when mixing calculated amounts of the contrast medium into the liquid resin manually. However, the almost perfect linearity of the measured mass density and voxel values in the scan indicates that the latter has been performed with appropriate accuracy, an external test would be favorable. Further work will compare attenuation in medical CT scanners with standardized iodine solutions prepared from non-ionic iodine compounds dissolved in deionized water to verify the iodine content in the printed samples.

Another limitation is the constancy of the supply of the resins used, as manufacturers can (and have) changed the chemical formulations of the resins without notice to the end user in order to improve the overall printing process and results. Therefore, when using a new batch, the resin properties must be thoroughly checked.

With the material developed in this work, calibration plates and quality control phantoms for contrast-enhanced mammography with exact iodine area weight are currently developed. Future envisaged developments include printing realistic tumor models in anthropomorphic phantoms, including appropriate “anatomical noise”, i.e., tissue background structures, for optimization and detectability studies in different 2D and 3D modalities, mainly in mammography and CT, including spectral and dual energy CT applications.

## Data Availability

Data are contained within the article.
